# Peritransplant Soluble CD30 as a Risk Factor for Slow Kidney Allograft Function, Early Acute Rejection, Worse Long-Term Allograft Function, and Patients' Survival

**DOI:** 10.1155/2017/9264904

**Published:** 2017-06-11

**Authors:** Andriy V. Trailin, Tetyana I. Ostapenko, Tamara N. Nykonenko, Svitlana N. Nesterenko, Olexandr S. Nykonenko

**Affiliations:** ^1^Department of Laboratory Diagnostics and General Pathology, State Institution “Zaporizhzhia Medical Academy of Postgraduate Education Ministry of Health of Ukraine”, 20 Winter Boulevard, Zaporizhzhia 69096, Ukraine; ^2^Department of Transplantology, Endocrine Surgery and Cardiovascular Surgery, State Institution “Zaporizhzhia Medical Academy of Postgraduate Education Ministry of Health of Ukraine”, Zaporizhzhia Regional Hospital, 10 Orikhiv Highway, Zaporizhzhia 69050, Ukraine; ^3^Institute of Cardiovascular Surgery and Transplantology, State Institution “Zaporizhzhia Medical Academy of Postgraduate Education Ministry of Health of Ukraine”, 20 Winter Boulevard, Zaporizhzhia 69096, Ukraine; ^4^Immunological Laboratory, Zaporizhzhia Regional Hospital, State Institution “Zaporizhzhia Medical Academy of Postgraduate Education Ministry of Health of Ukraine”, 10 Orikhiv Highway, Zaporizhzhia 69050, Ukraine

## Abstract

**Background:**

We aimed to determine whether serum soluble CD30 (sCD30) could identify recipients at high risk for unfavorable early and late kidney transplant outcomes.

**Methods:**

Serum sCD30 was measured on the day of kidney transplantation and on the 4th day posttransplant. We assessed the value of these measurements in predicting delayed graft function, slow graft function (SGF), acute rejection (AR), pyelonephritis, decline of allograft function after 6 months, and graft and patient survival during 5 years of follow-up in 45 recipients.

**Results:**

We found the association between low pretransplant serum levels of sCD30 and SGF. The absence of significant decrease of sCD30 on the 4th day posttransplant was characteristic for SGF, early AR (the 8th day–6 months), late AR (>6 months), and early pyelonephritis (the 8th day–2 months). Lower pretransplant and posttransplant sCD30 predicted worse allograft function at 6 months and 2 years, respectively. Higher pretransplant sCD30 was associated with higher frequency of early AR, and worse patients' survival, but only in the recipients of deceased-donor graft. Pretransplant sCD30 also allowed to differentiate patients with early pyelonephritis and early AR.

**Conclusions:**

Peritransplant sCD30 is useful in identifying patients at risk for unfavorable early and late transplant outcomes.

## 1. Introduction

Kidney transplantation is the optimal long-term treatment for most patients with end-stage chronic kidney disease [1]. However, death with functioning graft and chronic renal allograft dysfunction are the major obstacles to improve outcomes after kidney transplantation [1]. Infections, cardiovascular diseases, and cancer are the major causes of death with functioning graft [2–4], whereas acute rejection (AR) and impairment of initial kidney allograft function are among the major risk factors of chronic renal allograft dysfunction [5–7]. Thus, there is an urgent need to develop the reliable biomarkers to identify patients at higher risk of premature death or allograft loss. CD30 is a member of the tumor necrosis factor/nerve growth factor receptor superfamily [8], which is preferentially expressed by human T cell clones producing T helper (Th) type 2 cytokines, and can be released in a soluble form (sCD30) by activated cells [8, 9].

The ability of sCD30 to predict unfavorable outcomes after kidney transplantation has generated wide interest in the recent years; however, findings have been inconclusive. Many investigators have shown that higher pretransplant sCD30 is an indicator for the risk of acute kidney graft rejection [4, 9–11], although there are discrepancies regarding the ability of sCD30 to predict timing of AR occurrence [12] and thresholds of sCD30 [4, 5, 9, 10]. Moreover, a lot of studies [5, 13–22], including a meta-analysis by Chen et al. [21], failed to confirm that sCD30 pretransplant measurements could differentiate patients with and without AR. The relationship between pretransplant sCD30 and acute tubular necrosis (ATN) or delayed graft function (DGF) has been investigated in several studies. Some authors showed that ATN was characterized by low pretransplant levels of sCD30 [23]; however, other authors did not find such an association [17]. Pelzl and coworkers found that pretransplant sCD30 could differentiate patients with DGF and AR [23], whereas other groups [12, 17] did not confirm that finding. Contradictory data are reported regarding association of pretransplant levels of soluble CD30 and incidence of posttransplant infections [4, 24–26].

Many studies have examined the relationship between posttransplant sCD30 and AR. Some authors discovered an association of high early posttransplant sCD30 and risk of AR [14, 20, 23, 27], whereas others failed to show such a relation [18, 19, 22]. One group, which evaluated the association between pre- and posttransplant sCD30, reported that the absence of significant decrease of sCD30 posttransplant was characteristic for AR but not for DGF or uncomplicated course [20].

The association of higher pretransplant sCD30 [9, 10, 15, 16, 25, 28, 29] and posttransplant sCD30 [27, 28, 30–32] with increased risk of graft loss and worse graft survival was found in most published studies, including several large multicenter studies [9, 10, 32]. However, these results were not confirmed by other authors [5, 19, 33]. There have been only a few studies that examined the association of pretransplant levels of sCD30 with patient survival that yielded inconclusive results [4, 9, 25, 26, 34].

Several important questions remain unanswered. It is not entirely clear the significance of sCD30 dynamics from pre- to posttransplant [13, 20]. Also, different types of initial function were not considered [12, 17, 20]. Most studies included only recipients of cadaver graft [9, 10, 14, 25] or living-donor graft [5, 11, 28]. Sources describing the relationship of sCD30 with long-term kidney allograft function are few and contradictory [5, 19, 30, 31, 33, 35]. Finally, the effect of sCD30 on the survival of recipients is poorly described in the literature [4, 9, 25, 26, 34], which argues for the necessity of further study. We aimed to check the hypothesis that serum pre- and posttransplant sCD30 could identify recipients at high risk for unfavorable early and late transplant outcomes such as impaired initial function, AR, pyelonephritis, decline of allograft function after 6 months, and graft and patient survival during 5 years of follow-up.

## 2. Materials and Methods

### 2.1. Patients' Population

From February 2006 to July 2007, 54 consecutive Caucasian patients received a kidney allograft at Zaporizhzhia Transplantation Center. The criteria of inclusion to the study were the adult recipient of kidney allograft from related or deceased donor, male or female, written informed consent with participation in research, negative cross-match, and available follow-up data. The final population, who fulfilled the inclusion criteria, consisted of 45 patients, 26 males, and 19 females aged 21 to 56. All recipients received triple maintenance immunosuppressive therapy consisting of calcineurin inhibitor (cyclosporine A or tacrolimus), antiproliferative agent (mycophenolate mofetil (MMF) or azathioprine), and steroid. Eighty percent of patients received anti-CD25 antibodies for induction. All participants gave written informed consent. This research was approved by the local ethics committee and carried out in accordance with the ethical standards laid down in the Declaration of Helsinki and the Declaration of Istanbul.

### 2.2. Laboratory Methods

Donor-recipient blood group matching was identical in all patients. All included patients had negative results of microlymphocytotoxicity cross-match at the time of transplantation (<5%). Information on HLA status of recipients and donors and panel-reactive antibody (PRA) status of recipients was not available for most patients and thus not presented. Serum for the analysis of sCD30 was collected on the day of transplantation prior to introduction of immunosuppressive therapy and on the 4th day posttransplant. For measuring, sCD30 serum had been frozen and stored at minus 40°С. We evaluated sCD30 concentrations at 3 months after the last patient was discharged from the hospital. We assessed serum sCD30 concentrations in pretransplant samples from all enrolled persons and in “day 4”-sera from 33 selected patients. These 33 patients consisted of 23 persons who experienced slow graft function (SGF) and DGF, as well as AR or pyelonephritis within the first 3 months and 10 persons with uncomplicated course. Serum sCD30 content was determined using the commercially available ELISA kit of Bender MedSystems (Vienna, Austria) according to the manufacturer's protocol. We measured the absorbance using a plate reader from Tecan Sunrise (Austria). As indicated by the manufacturer, the intra-assay variance of this assay is 4.1% and the interassay variance is 5.6%. sCD30 levels reported by the manufacturer for healthy population ranged from 17.5 U/mL to 130.7 U/mL, with a mean level of 38.7 U/mL.

### 2.3. Risk Factors and Outcomes Examined

In this study, we assessed an independent value of serum sCD30 pretransplant and early posttransplant in predicting adverse posttransplant outcomes. Archival patient records and outpatient cards were used to obtain more information on major risk factors, evolution of allograft function, and outcomes.

Pretransplant variables included recipient's age; gender; weight; cause of ESRD; dialysis modality and duration; presence of chronic arterial hypertension which is defined as a regular intake of antihypertensive drugs; previous transplants, pregnancies, and transfusions; use of induction therapy; and type of initial immunosuppression. Donor variables included source (living or deceased), age, and cause of death for deceased donors (stroke or traumatic brain injury); heart-beating versus non-heart-beating deceased donors; cold ischemia time; and second warm ischemia time. We also obtained the information related to posttransplant course as follows: type of initial graft function, serum creatinine at discharge from the hospital, episodes of acute rejection and their characteristics, episodes of pyelonephritis, date of graft failure (defined as the date of reinitiation of dialysis), and date of death.

We classified initial allograft function as follows: immediate graft function (IGF), SGF, that is, serum creatinine on day seven ≥300 *μ*mol/L without evidence of AR or pyelonephritis, or DGF, that is, requirement for dialysis in the first week posttransplantation without evidence of AR or pyelonephritis. For linear regression analysis, the initial allograft function was classified as follows: immediate function (0 points) or impaired function, that is, SGF (1 point), or DGF (2 points). AR was defined by the need for treatment, with or without biopsy confirmation. AR episodes were classified as very early (0–7th day), early (the 8th day–6 months), and late (>6 months). Two of twelve (17%) of acute rejection episodes were confirmed by renal biopsy. For linear regression analysis, episodes of AR were classified as follows: absence of AR (0 points), AR successfully treated by steroid therapy (1 point), and steroid-resistant AR (2 points). We defined pyelonephritis by characteristic symptoms, a urine sediment analysis, and a urinary culture test. Episodes of pyelonephritis were classified as early (the 8th day–2 months) or late (>6 months).

Patients, enrolled to this study, were followed for five years until death/return to dialysis or until December 2012. In the course of the follow-up period, we recorded serum creatinines measured in all patients at months 1, 3, 6, and 12 and annually until graft failure/death or last follow-up. These serum creatinines were used to estimate the glomerular filtration rate (eGFR) with the Chronic Kidney Disease Epidemiology Collaboration equation. We calculated slopes in eGFR during a period of 1–6 months (mL/min/1.73 m^2^/month) and 6 months–5 years (mL/min/1.73 m^2^/year) for each patient, having at least three eGFR measurements during each period, by the linear mixed effects model with varying intercepts and slopes. We determined the proportion of patients having an eGFR slope ≥−5 mL/min/1.73 m^2^/year and the proportion of patients having an eGFR drop of ≥25% from 6 months, since both measures indicate progressive loss of kidney function [36]. During the follow-up, a total of four deaths with functioning graft occurred and six grafts failed. For six patients, who returned to dialysis, we imputed a GFR of 10 mL/min/1.73 m^2^. The endpoints of the study were DGF; SGF; episodes of AR and pyelonephritis; the eGFR at 1, 3, and 6 months and annually up to 5 years; the slope of eGFR_1–6 months_ and the slope of eGFR_6 months–5 years_; the certain drop in eGFR of ≥25% from 6 months; the rapid decline in eGFR of 5 mL/min/1.73 m^2^/year; allograft failure; and death with functioning graft.

### 2.4. Statistics

Normally distributed data are expressed as mean ± SD; Pearson's correlation coefficient (*r*) was calculated. Continuous nonparametric data are expressed as median (interquartile range); for comparison, we used the Kruskal-Wallis test with post hoc comparisons, Mann–Whitney's test, or Wilcoxon's test when appropriate; the Spearman's correlation coefficient (rho) was calculated. Frequency data are expressed as percentages, and for comparison, we applied the chi-square test. Relative posttransplant changes of sCD30 levels in subgroups of patients were expressed as delta percentages and compared as appropriate. To identify predictors of eGFR at different times posttransplant and those of eGFR slope, multiple linear regression with backward stepwise selection was used. We used logistic regression analysis to identify independent predictors of SGF, AR, a certain drop in eGFR, and a rapid decline in eGFR. In addition, we calculated areas under the receiver-operating characteristic curves (AUC) to assess the capability of sCD30 and other variables of interest to discriminate patients with SGF and early AR from those with an uncomplicated course, as well as, between early AR and early pyelonephritis. Cutoffs were derived from the ROC curve to yield empirical optimal sensitivity and specificity. For some analyses we used previously determined by Susal and coworkers clinically relevant threshold for pretransplant sCD30 of 100 U/mL [9]. Cumulative survival was calculated by the Kaplan-Meier method and compared by log-rank test. We used Cox regression to analyze the effect of risk factors on survival. Statistica 7.0 (StatSoft Inc., Tulsa, USA) and SPSS (version 19.0 SPSS Inc., Chicago, USA) packages were used for statistical analyses. Statistical significance was set at *P* < 0.05.

## 3. Results

### 3.1. Baseline Characteristics of Patients

Patients' baseline characteristics are depicted in Table 1. The majority of the patients received the first kidney graft from a heart-beating deceased donor. Eighty percent of the patients received anti-CD25 antibodies for induction immunosuppression. Some patients had previous kidney transplants, transfusions, or pregnancies (Table 1).

### 3.2. sCD30 Levels and Their Correlation with Clinical Variables at Baseline

Table 2 outlines pre- and posttransplant levels of sCD30 in different subsets of patients. Pretransplant serum sCD30 levels ranged from 9.1 to 169.6 U/mL with a median value of 49.5 U/mL, and they were higher in younger patients (*r* = −0.373, *P* = 0.012) as well as in the recipients of living-donor graft (rho = −0.312, *P* = 0.037, Table 2). Pretransplant sCD30 levels were not associated significantly with pregnancies, transfusions, and previous transplantations. Posttransplant sCD30 levels decreased in most patients and ranged from 8.8 to 56.7 U/mL with a median value of 23.1 U/mL, and they were not influenced by baseline recipients and donors' characteristics. Pre- and posttransplant sCD30 levels significantly correlated with each other (*r* = 0.535, *P* = 0.001). The significant decrease of sCD30 posttransplant (*P* < 0.01) was observed irrespectively of donor age. However, the median decline was more pronounced in the recipients of grafts from younger (−54.46%) than from older (−27.31%) donors (*P* = 0.019). The decrease was significant only in recipients of deceased-donor graft (*P* < 0.001). sCD30 declined significantly posttransplant both in males and in females (*P* < 0.001), and the median decline was more evident in males (−52.68%) than in females (−24.02%) (*P* = 0.053). The significant decrease was absent in women with previous pregnancies (*P* = 0.068). The decrease was significant only in patients on pretransplant hemodialysis (*P* < 0.001) versus those on peritoneal dialysis (Table 2). We observed significant decrease of posttransplant sCD30 levels only in patients (*N* = 27) who received anti-CD25 antibodies for induction therapy (*P* < 0.001).

### 3.3. Initial Graft Function, Acute Rejection, Pyelonephritis, and Predictive Variables

**Figure 1:**
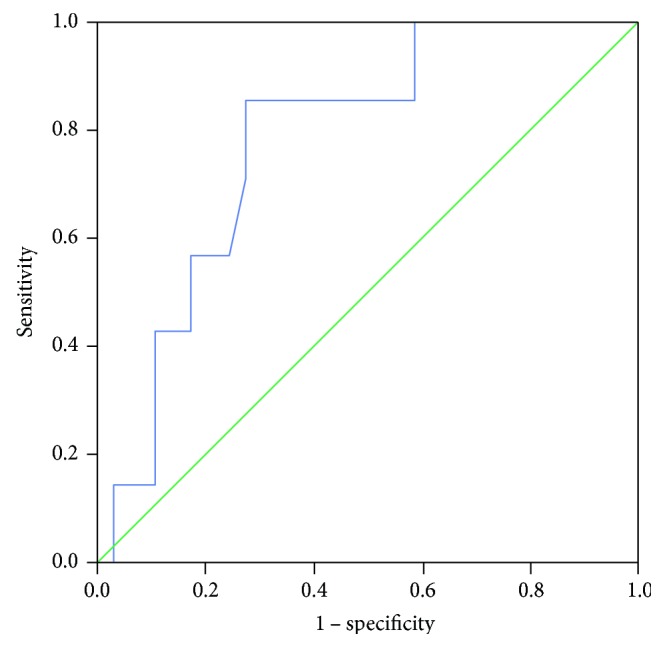
ROC curve for pretransplant serum sCD30 to predict SGF. AUC (95% confidence intervals) is 0.781 (0.613–0.948), *P* = 0.023. ROC: receiver-operating characteristic; AUC: area under the curve; sCD30: soluble CD30; SGF: slow graft function.

Outcomes of early and late posttransplant period are summarized in Table 3. Pretransplant levels of sCD30 did not differ significantly between patients with IGF (*N* = 29): 54.8 (36.9–72.6) U/mL and DGF: 46.6 (36.2–64.2) U/mL (*P* > 0.05). However, sCD30 levels were lower (*P* = 0.046) in patients who experienced SGF: 27.3 (21.7–40.1) U/mL, compared to those in the IGF group. Logistic regression showed that lower pretransplant sCD30 was a weak but significant predictor of SGF (OR = 0.95, CI: 0.90–1.00, *P* = 0.046). None of other variables were associated with SGF or DGF. Lower pretransplant sCD30 exhibited fair discriminatory power in predicting the SGF (AUC = 0.781, CI: 0.613–0.948, *P* = 0.023). We derived the cut-off value of 60.1 U/mL from the ROC curve (see Figure 1), where sCD30 exhibited the highest sensitivity for SGF (100%). Posttransplant sCD30 levels did not differ according to type of initial graft function (*P* = 0.551, Table 4). Levels of sCD30 decreased significantly on the 4th day posttransplant in patients with IGF and DGF (Table 4) but remained on the pretransplant level in the SGF group.

Overall, 27% of patients had episodes of very early, early, and late AR during follow-up (Table 3). Two cases of late AR diagnoses were confirmed histologically, and we found acute/active antibody-mediated rejection. All episodes of very early AR were effectively treated with steroids, whereas two cases of early AR and two cases of late AR were steroid-resistant. All patients with steroid-resistant AR had unfavorable prognosis: three of them returned to dialysis in 7, 11, and 16 months, respectively, and the fourth patient died in 13 months due to pulmonary tuberculosis.

**Figure 2:**
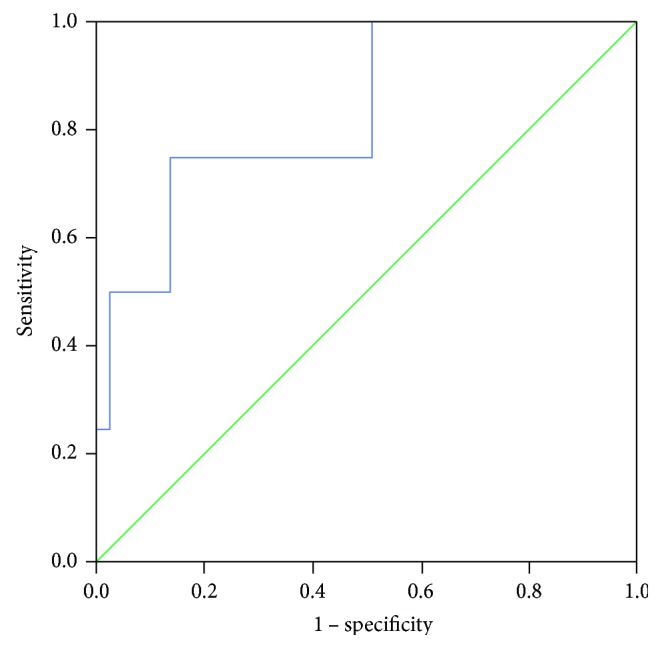
ROC curve for pretransplant serum sCD30 to predict early AR after cadaver kidney transplantation. AUC (95% confidence intervals) is 0.829 (0.613–1.000), *P* = 0.033. ROC: receiver-operating characteristic; AUC: area under the curve; sCD30: soluble CD30; AR: acute rejection.

Pregnancies predicted occurrence of AR at any time (OR = 16.00, CI: 1.46–174.90, *P* = 0.019). Pregnancies (OR = 12.67, CI: 1.21–133.03, *P* = 0.029) and cold ischemia time (OR = 1.48, CI: 1.00–2.20, *P* = 0.046) predicted early AR. Pretransplant levels of sCD30 did not differ significantly between patients without AR (*N* = 33): 48.3 (30.8–70.6) U/mL, very early AR: 56.1 (51.0–60.2) U/mL, early AR: 83.7 (57.0–130.4) U/mL, and late AR: 36.9 (17.1–40.9) U/mL (*P* > 0.05). When we randomized patients according to the median pretransplant sCD30 (49.5 U/mL), the frequency of early AR was higher in the high sCD30 group (31.8% versus 8.7%) with tendency to significance (*P* = 0.053). Pretransplant sCD30 significantly predicted the occurrence of only early AR and exclusively in the recipients of cadaver grafts (OR =1.05 (1.00–1.09), *P* = 0.035). In multivariate analysis, both pregnancies and higher pretransplant sCD30 predicted the early AR in the recipients of deceased-donor graft: OR = 23.31, CI: 1.05–517.08 (*P* = 0.039) and OR = 1.05, CI: 1.00–1.09 (*P* = 0.033), respectively. The ROC curve on Figure 2 shows the ability of pretransplant sCD30 to predict early AR in this subgroup: AUC = 0.829, CI: 0.613–1.000, *P* = 0.033. sCD30 exhibited good specificity (85.7%) and sensitivity (75%) at the cut-off value of 70.6 U/mL. Posttransplant sCD30 levels did not differ according to the occurrence and timing of AR (*P* > 0.05, Table 4). Levels of sCD30 decreased significantly on the 4th day posttransplant in patients without AR, and with very early AR, but the decrease was not significant in patients with early and late AR (Table 4).

Five (11.1%) and two (4.4%) patients had early and late pyelonephritis, respectively (Table 3). Patients with and without early pyelonephritis did not differ significantly in their sCD30 levels pretransplant: 27.3 (21.7–59.9) U/mL versus 50.2 (35.1–72.1) U/mL, *P* = 0.220, and in their levels of sCD30 posttransplant as well (Table 4, *P* = 0.119). sCD30 significantly decreased posttransplant only in patients without early pyelonephritis (Table 4). Development of early pyelonephritis was predicted by the duration of pretransplant dialysis (OR =1.05, CI: 1.01–1.10, *P* = 0.010) and by SGF (OR = 28.00, CI: 2.41–325.24, *P* = 0.006), whereas only SGF was significant in multivariate analysis (OR = 26.19, CI: 1.64–417.61, *P* = 0.017).

**Figure 3:**
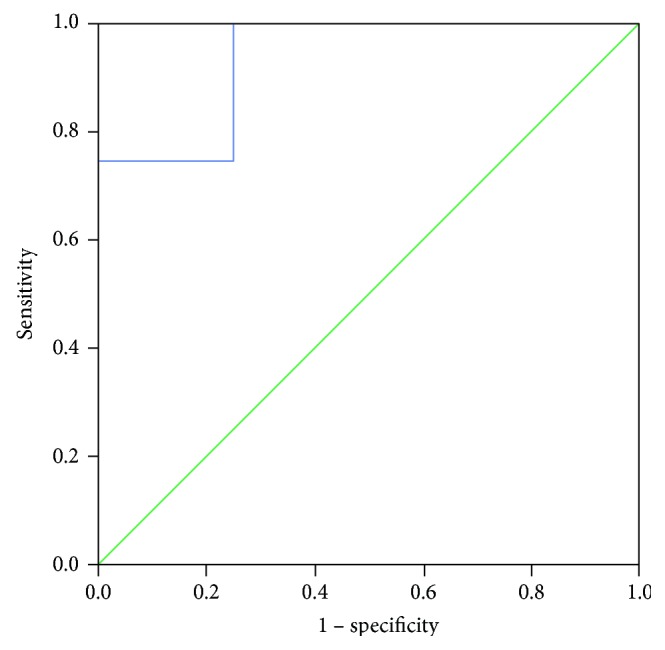
ROC curve for pretransplant serum sCD30 to discriminate between early AR and pyelonephritis. AUC (95% confidence intervals) is 0.938 (0.762–1.000), *P* = 0.043. ROC: receiver-operating characteristic; AUC: area under the curve; sCD30: soluble CD30; AR: acute rejection.

Pretransplant sCD30 levels were significantly lower in patients with subsequent development of early pyelonephritis compared with those who experienced early AR (*P* = 0.040).  Figure 3 shows the ROC curve for ability of serum sCD30 to discriminate recipients with early pyelonephritis and early AR. The analysis yielded an AUC of 0.938, CI: 0.762–1.000, indicating excellent prognostic value (*Р* = 0.043), and at the cut-off value of 65.3 U/mL, sCD30 displayed the highest specificity (100%) and good sensitivity (75%).

### 3.4. The Evolution of Allograft Function and the Predictive Variables

Median serum creatinine at discharge was 187 (136–242) *μ*mol/L. During the first 6 months, allograft function gradually improved. The median slope of eGFR_1–6 months_ was 1.29 mL/min/1.73 m^2^/month, and its magnitude was correlated only with episodes of early AR (rho = −0.313, *P* = 0.037). After 6 months, the median annual slope was negative: −0.93 mL/min/1.73 m^2^/year. Only two patients demonstrated rapid decline in eGFR of >−5 mL/min/1.73 m^2^/year from 6 months to 5 years, and this decline was correlated with episodes of AR at any time and late AR: rho = 0.298, *P* = 0.046, and rho = 0.313, *P* = 0.037, respectively. Nine patients (20%) had lost ≥25% of eGFR by the end of the 5th year that correlated only with previous transplantations (rho = 0.310, *P* = 0.041). Pretransplant and posttransplant sCD30 did not demonstrate association with slopes in eGFR, as well as with their steepness and magnitude.

Predictive variables for eGFR at different times are presented in Table 5. Impairment of initial graft function predicted lower eGFR at 1, 3, and 6 months in multivariate analysis. AR demonstrated negative impact on eGFR at 6 months. Previous transplants predicted lower eGFR at 3 years. Lower pretransplant sCD30 was not independently associated with lower eGFR at 3 months; however, lower sCD30 independently predicted lower eGFR at 6 months (Table 5). We observed significantly (*Р* < 0.05) lower eGFR at 1, 3, and 6 months in patients with pretransplant sCD30 < 100 U/mL: 42 (27–55), 50 (41–65), and 54 (43–63) mL/min/1.73 m^2^, respectively, versus 62 (58–64), 68 (65–72), and 69 (64–74) mL/min/1.73 m^2^, respectively, in patients (*N* = 4) with pretransplant sCD30 ≥ 100 U/mL. Lower sCD30 on the 4th day posttransplant independently associated with lower eGFR at 2 years (Table 5).

### 3.5. Patient and Graft Survival and Predictive Variables

**Figure 4:**
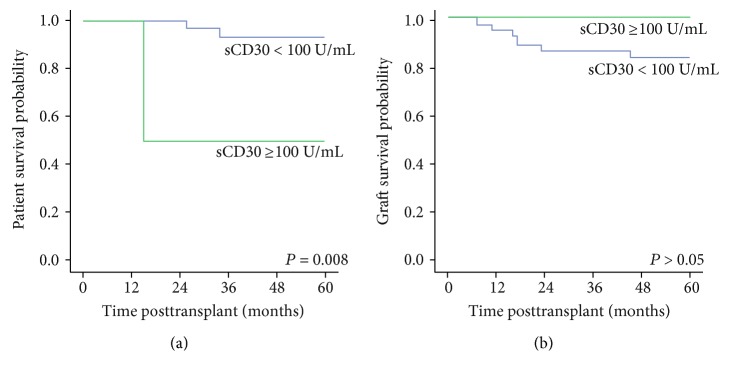
Impact of pretransplant sCD30 on patient survival (a) and death-censored graft survival (b) after cadaver kidney transplantation (Kaplan–Meier estimates). *P* values are calculated with the log-rank test. sCD30: soluble CD30.

During the follow-up, a total of four deaths with functioning graft occurred. Causes of deaths were stroke, pulmonary tuberculosis, myocardial infarction, and primary renal cell carcinoma. The 5-year patient survival rate was nonsignificantly better in patients with shorter pretransplant dialysis (100% versus 82.6%, *P* = 0.061). Cox multivariable analysis showed that duration of dialysis had an impact on patients' survival: HR = 1.029 (1.010–1.049), *P* = 0.003. Pretransplant sCD30 did not influence patients' survival in the whole cohort (data not shown); however, 5-year survival of the recipients of deceased-donor graft with pretransplant sCD30 ≥ 100 U/mL (*N* = 2) was 50% versus 94.6% in the group (*N* = 37) with pretransplant sCD30< 100 U/mL (*P* = 0.008, Figure 4). Cox analysis confirmed that high pretransplant sCD30 significantly predicted worse 5-year survival rate in the recipients of cadaver graft: HR 1.04 (1.00–1.08), *P* = 0.021.

Six patients lost their grafts because of allograft dysfunction, in three of whom acute antibody-mediated rejection (*N* = 2) and thrombosis of allograft vein were confirmed histologically. Three other patients lost their grafts due to chronic allograft dysfunction. The 5-year graft survival rate was 58.3% in patients who experienced AR at any time versus 97.0% in nonrejecting patients (*P* = 0.002). Patients with early AR also exhibited worse allograft survival: 50.0% versus 90.2 in nonrejectors (*P* = 0.002). In Cox regression, only episodes of AR at any time and early AR predicted allograft loss: HR = 18.18 (2.11–156.33), *P* = 0.008, and HR = 9.72 (1.71–55.25), *P* = 0.010, respectively. sCD30 pretransplant had no impact on 5-year graft survival in the whole group, which constituted 100% in patients with pretransplant sCD30 ≥ 100 U/mL versus 85.4% in the group with pretransplant sCD30 < 100 U/mL (*P* > 0.05), HR = 0.99 (CI: 0.97–1.02), *P* > 0.05. Pretransplant sCD30 also had no impact on graft survival in the recipients of cadaver graft (Figure 4), which was 100% in patients with pretransplant sCD30 ≥ 100 U/mL versus 83.8% in the group with pretransplant sCD30 < 100 U/mL (*P* > 0.05), HR1.00 (0.97–1.03), *P* > 0.05.

sCD30 levels on the 4th day posttransplant had no significant impact on patients' and graft survival in the whole group and in the recipients of cadaver graft as well (data not shown).

## 4. Discussion

The search for noninvasive biomarkers of nonfavorable kidney transplant outcomes is an urgent need. In this study, we found that higher pretransplant sCD30 predicted higher frequency of early AR, and worse patients' survival, but we observed this particular effect only in the recipients of deceased-donor graft. Lower pretransplant serum level of sCD30 was associated with SGF. The absence of significant decrease of sCD30 on the 4th day posttransplant was characteristic for SGF, early and late AR, and early pyelonephritis. Pretransplant sCD30 also allowed to differentiate patients with early pyelonephritis and early AR. Lower pretransplant and posttransplant sCD30 predicted worse allograft function at 6 months and 2 years, respectively.

Our first finding is that higher pretransplant sCD30 predicts episodes of only early AR, and this effect is observed only in the recipients of deceased-donor graft. Apparently, the immune system of recipients with higher sCD30 is more activated [29]; hence, patients are prone to reject more immunogenic graft of inferior quality, which is typical for cadaver kidney [37]. Prognostic role of sCD30 for AR was established earlier in several studies of cadaver kidney transplantation [4, 11], including large multicenter trials [9, 10, 14]. Some authors showed the ability of pretransplant sCD30 to predict AR in living-donor kidney transplantation [11] and in a mixed population of patients who received allografts from deceased and living donors [16]. However, as in our study, other authors did not observe association between pretransplant [5, 22] and early posttransplant [22] sCD30 levels and frequency of AR in live-related renal transplant program. Our observation confirms and completes literature data that show the value of pretransplant assessment of sCD30 in the prediction of kidney graft rejection [4, 9–11]. Nevertheless, in our study, higher pretransplant sCD30 significantly predicted AR in a time-dependent fashion: within the period from the 8th day to 6 months. Particular time of occurrence of AR is often associated with different clinical and histopathological characteristics [7, 38]. Although diagnosis of early AR was clinical and not histological, indeed, episodes of early AR shared several features of antibody-mediated rejection. Characteristically, they were predicted by history of pregnancies, 50% of them were steroid-resistant and they significantly worsen allograft survival [7, 38, 39]. Literature data also show that higher levels of sCD30 are associated with Th2-mediated immune response [8, 9] and higher risk of antibody-mediated rejection [40–42]. Even though we did not have data on PRA status of our patients, pretransplant sCD30 levels were not associated with such clinical markers of sensitization as pregnancies, transfusions, and previous transplantations. This finding indirectly confirms existing data [9, 29, 40] that T cell activation marker sCD30 is a good indicator of immunological risk apart of antibody sensitization. Our results also imply that pretransplant sCD30 can help in risk stratification of patients without classical signs of sensitization that was suggested earlier by other authors [43]. Pretransplant sCD30 has high specificity and sensitivity in predicting early AR in the recipients of deceased-donor graft at a threshold of 70.6 U/mL in our study. Predictive values of sCD30 for AR reported by other authors were in average higher and ranged from 100 U/mL to 400 U/mL [4, 5, 9, 10]. However, they were not time-specific [9, 10], or were established in patients who received kidney transplant from living-donors [5], or from mixed donor population [4]. Several groups showed that sCD30 pretransplant measurements could not differentiate patients with and without AR, but the authors disregarded timing of AR [13, 17] or included into their study only transplantations from living donors [5]. Moreover, negative results, obtained by some authors, can be attributed to use of potent immunosuppression (e.g., tacrolimus-based immunosuppression or regimen with rituximab for induction) [18].

Since Altermann and coworkers [44] found high degree of variation of sCD30 values pretransplant, which limits its implementation as a prognostic marker, we also assessed this biomarker on the 4th day posttransplant. sCD30 has decreased posttransplant in most patients, which is in agreement with previously published results [14, 17, 20, 32]. Like several other studies [18, 19, 22], our study could not reproduce results showing association of higher sCD30 on the 3rd–5th days posttransplant with ongoing or impending AR [17, 23, 27]. One of the reasons for this was that posttransplant levels of sCD30 in our cohort were generally lower than those reported in the literature [13, 17]. Furthermore, the decrease of sCD30 after transplantation was associated in our patients with the use of anti-CD25 antibodies. Most patients in the study (80%) received induction therapy; hence, they were at lower risk for AR [45]. Nevertheless, we suggest that further investigations are necessary to evaluate the predictive value of posttransplant sCD30. We have also shown that patients, in whom sCD30 did not fall significantly posttransplant, developed more frequently early and late AR. The absence of decrease of serum sCD30 posttransplant in patients with impending AR was described earlier by other authors [20]. The decrease of sCD30 after transplant in our study was absent or was less significant in females, especially in those with pregnancies in anamnesis, in the recipients of the kidney from older donors, and in recipients of living-donor graft. As highlighted by several studies [39, 46, 47], all these factors might provoke sensitization and, consequently, higher incidence of AR. In addition, we found a link between previous pregnancies and AR, as well as between previous transplants and worse function at 3 years, which confirm negative consequences of presensitization in our patients' cohort. Since the use of anti-CD25 antibodies led to significant decrease of sCD30 after transplantation, we suggest that patients with high pretransplant sCD30 can profit from the use of anti-CD25 antibodies or other intense immunosuppressive therapy directed on T cells, which was considered earlier by other authors [9, 15].

Our second significant result is that lower pretransplant sCD30 is associated with higher incidence of SGF but not with DGF. Pretransplant sCD30 showed fair power to discriminate patients with SGF. We found no published data on the relationship between pretransplant sCD30 and SGF, although the absence of association between higher pretransplant sCD30 and DGF was reported previously [14, 17]. Association of pretransplant sCD30 with SGF seems to be important considering that SGF shares ethiopathogenesis with DGF, and both conditions have worse prognosis compared with IGF [6]. Lower pretransplant sCD30 could be a marker of those risk factors that could trigger SGF in our patients' cohort, like use of deceased-donor graft. Patients with SGF also demonstrated the absence of significant decline of sCD30 on the 4th day posttransplant. Accordingly, the dynamics of sCD30 from pre- to posttransplant might be used to help diagnose SGF, since this diagnosis is often subjective [6]. It is also worth mentioning that several authors have postulated the participation of T lymphocytes in ischemic-reperfusion injury after kidney transplantation [48, 49]. They suggest that impairment of initial function is characterized by a prevalent Th1 phenotype within the graft [48], for which lower sCD30 is more characteristic, as evidenced by the literature [8]. Thereby, our data support the immune paradigm of ischemic reperfusion injury [49].

We further demonstrated that sCD30 significantly decreased posttransplant in patients without pyelonephritis within the period from the 8th day to 2 months, whereas the decrease was not significant in a subset of recipients who experienced pyelonephritis. Early pyelonephritis in our patients was also associated with previous SGF in multivariate analysis. The association of urinary tract infections with delayed graft function was shown earlier by some authors [50]. Thus, the absence of decline of sCD30 posttransplant and subsequent development of pyelonephritis could simply reflect the effects of SGF itself. Additionally, the absence of decline of sCD30 posttransplant can mirror ongoing Th2 activity and thereby lower activity of Th1 lymphocytes, which can predispose patients to urinary tract infections [26, 51, 52]. We also managed to show that pretransplant serum sCD30 could differentiate patients with the risk of early AR (high sCD30) or early pyelonephritis (low sCD30).

We report here the association of lower pretransplant and early posttransplant sCD30 levels with lower GFR at 6 months and 2 years, respectively. Impaired initial function and episodes of AR are also shown to be independent predictors of lower eGFR at 6 months, and so, can be used, along with pretransplant sCD30, in predictive models. As long as lower pretransplant sCD30 was associated with transplantation from deceased donor and SGF, it is logical to assume that suboptimal graft function at 6 months also might be linked to these conditions, which is supported by the literature [1, 6]. Lower posttransplant levels of sCD30 in individual patients, in its turn, can be explained by lower pretransplant levels of this biomarker, as our analysis show moderate correlation between them. On the contrary to our results, several groups found the association of higher pre- and posttransplant sCD30 with worse allograft function [5, 30, 31, 35] up to 2 years [35] and 3 years [5] or reported the absence of such a link [19, 33]. Thus, more studies are needed to elucidate the relationships between sCD30 and late allograft function.

Similar to several earlier studies [5, 19, 33], we have found no association of pre- or posttransplant sCD30 levels and graft survival. However, other groups found the link between higher pretransplant sCD30 [9, 10, 15, 16, 25, 28, 29] and posttransplant sCD30 [28, 30–32] and increased risk of graft loss or worse graft survival. These discrepancies might be due to relatively smaller-sized and more heterogeneous cohort in our study, and also because of different threshold used. We also noted that in papers cited above, posttransplant sCD30 was measured from the 30th day to 1 year posttransplant, comparing to the 4th day in the present research. We suppose, as well, that the absence of the effects of sCD30 serum levels on allograft survival could be related to the use of anti-CD25 antibodies in most of our patients. Previously, Kovac and coauthors concluded that immunosuppression with anti-CD25 antibodies and a triple CsA-based maintenance regimen could prevent the negative effect of higher pretransplant sCD30 on kidney graft function at 3 years after transplantation [33].

Our last finding is the association of higher levels of pretransplant sCD30 with worse survival of the recipients of deceased-donor graft. This result, although obtained on a very small sample, is in line with published data on the association of higher sCD30 levels and worse patients' survival after cadaver kidney transplantation [9, 25]. In our study, three deceased-donor kidney recipients died from cerebral hemorrhage, tuberculosis, and cancer, respectively. As highlighted in the literature, cerebral infarction [53], tuberculosis [54], and cancer [55] are characterized by a shift of Th1/Th2 balance toward Th2 and downregulation of cell-mediated immunity. High sCD30, as a Th2 marker, may reflect the risk of these complications, which is supported by the literature [24, 56]. The association of higher pretransplant sCD30 with increased frequency of early AR can also be taken into account, because AR itself is an independent risk factor for malignancy and coronary heart disease after kidney transplantation [2, 3].

Our study has several limitations. It was a single-center study carried out on a small group of Caucasian patients. Therefore, in a study with a larger cohort, some nonsignificant results may become significant. Some significant results, which are although in agreement with literature reports, need to be interpreted with caution. For the same reason, the cut-off values calculated for sCD30 should be considered preliminary, and they also require validation in an independent cohort. We had no data on PRA or donor-specific antibodies, so we had to make conclusions about the possible effects of sensitization only on the basis of clinical data. Also, not all acute rejections and causes of allograft loss were biopsy-proven and classified according to the Banff criteria. Further studies are needed to approve that peritransplant sCD30 serum levels can be a reliable marker of unfavorable transplant outcomes.

## 5. Conclusions

The results of the present study demonstrate the association between low and high pretransplant and posttransplant serum sCD30 and unfavorable short- and long-term outcomes after kidney transplantation. Higher pretransplant sCD30 portends a higher risk of early AR and worse patients' survival but only in the recipients of deceased-donor graft. Low pretransplant sCD30 is characteristic for SGF. The dynamics of sCD30 from pre- to posttransplant is important: sCD30 does not decrease significantly posttransplant in patients with SGF, early and late AR, and early pyelonephritis. Pretransplant sCD30 also allows to differentiate patients with early AR or SGF and those with uncomplicated early posttransplant course as well as to differentiate patients with early AR and early pyelonephritis. Lower pretransplant and posttransplant sCD30 are associated with suboptimal kidney graft function at 6 months and 2 years, respectively. We conclude, therefore, that peritransplant sCD30 have promise to improve individual risk prediction of unfavorable kidney transplant outcomes.

## Figures and Tables

**Table 1:** Donor and recipient population demographics.

Parameters	Recipients, *n* = 45
Recipients: age at baseline, years	41 ± 10^∗^
Gender, male versus female, *n* (%)	26 (57.8)/19 (42.2)^†^
Weight, kg	73.0 ± 15.7
Cause of ESRD, *n* (%)	
Glomerulonephritis	37 (82.3)
Polycystic kidney disease	4 (8.9)
Pyelonephritis/interstitial nephritis	2 (4.4)
Diabetic nephropathy	1 (2.2)
Congenital urological anomaly	1 (2.2)
Dialysis modality: hemodialysis versus peritoneal dialysis, *n* (%)	42 (93.3)/3 (6.7)
Duration of dialysis treatment, months	27.3 ± 24.9
Treated hypertension at baseline, *n* (%)	23 (51.1)
Previous transplants, *n* (%)	2 (4.4)
Previous blood transfusions, *n* (%)	8 (17.8)
Previous pregnancies, *n* (%)	5 (11.1)
Calcineurin inhibitor used initially, CsA versus tacrolimus, *n* (%)	44 (97.8)/1 (2.2)
Anti-CD25 antibodies use, *n* (%)	36 (80.0)
Donors: deceased versus living related, *n* (%)	21 (77.8)/6 (22.2)
Age (years) deceased	37 (30–47)
Living related	50 (45–58)
Cause of deceased donor death, stroke versus brain injury, *n* (%)	11 (52.4)/10 (47.6)
Heart-beating versus non-heart-beating deceased donors, *n* (%)	17 (81.0)/4 (19.0)
CIT (hours) deceased/living related donors	15 (13–18)/1 (1–1)^‡^
Second warm ischemia time, minutes	20 (18–23)

ESRD: end-stage renal disease; CsA: cyclosporine A; *n*: number of patients investigated; CIT: cold ischemia time. ^∗^Mean ± standard deviation; ^†^numbers (percentages); ^‡^median (interquartile range).

**Table 2:** Median sCD30 levels according to demographic characteristics of kidney allograft recipients at baseline.

Patient's characteristics	*n*	Pretransplant sCD30	*P* value	*n*	Posttransplant sCD30	*P* value
Age	21–40 (*n* = 23)	54.8 (36.9–96.6)^∗^	0.151	21–39 (*n* = 17)	21.4 (15.7–40.1)	0.817
	>40 (*n* = 22)	42.0 (30.8–60.2)		>39 (*n* = 16)	23.3 (14.8–37.5)	
Gender	Female (*n* = 20)	42.1 (29.1–71.2)	0.595	Female (*n* = 17)	26.6 (21.2–39.7)	0.260
	Male (*n* = 25)	51.6 (36.2–64.2)		Male (*n* = 16)	18.4 (12.9–38.8)	
Donor source	Living (*n* = 6)	85.6 (40.9–159.9)	0.038	Living (*n* = 4)	39.4 (30.3–45.0)	0.133
	Deceased (*n* = 39)	48.3 (27.3–64.2)		Deceased (*n* = 29)	21.4 (13.4–37.6)	
Donor age	17–38 (*n* = 22)	54.8 (36.2–70.6)	0.960	17–39 (*n* = 17)	21.4 (13.4–40.1)	0.709
	>38 (*n* = 23)	45.0 (29.7–79.5)		>39 (*n* = 16)	25.1 (15.9–38.7)	
Primary disease	GN (*n* = 37)	51.0 (30.8–70.7)	0.782	GN (*n* = 28)	22.2 (14.6–38.7)	0.643
	non-GN (*n* = 8)	45.8 (38.8–78.4)		non-GN (*n* = 5)	23.5 (16.1–45.3)	
Dialysis modality	HD (*n* = 42)	50.2 (30.8–70.7)	0.814	HD (*n* = 30)	21.4 (13.4–37.6)	0.075
	PD (*n* = 3)	40.9 (40.1–88.5)		PD (*n* = 3)	44.8 (34.0–52.9)	
Dialysis duration	≤18 m (*n* = 22)	50.6 (32.2–71.7)	0.711	<18 m (*n* = 16)	30.3 (19.2–42.5)	0.146
	>18 m (*n* = 23)	48.3 (30.8–70.6)		≥18 m (*n* = 17)	19.9 (12.4–28.0)	
Pregnancies	Yes (*n* = 5)	60.2 (40.9–70.7)	0.661	Yes (*n* = 4)	27.7 (16.5–45.4)	0.730
	No (*n* = 40)	48.9 (31.5–71.1)		No (*n* = 29)	23.1 (15.7–39.7)	
Transfusions	Yes (*n* = 8)	47.5 (42.0–58.2)	0.988	Yes (*n* = 7)	23.5 (15.7–39.7)	0.682
	No (*n* = 37)	51.0 (27.3–72.6)		No (*n* = 26)	22.2 (13.4–40.1)	
Previous transplants	Yes (*n* = 2)	57.6 (51.0–64.2)	0.618	Yes (*n* = 2)	20.6 (13.4–27.8)	0.689
	No (*n* = 43)	48.3 (30.8–71.7)		No (*n* = 31)	23.1 (15.7–40.1)	
Anti-CD25 antibodies	Yes (*n* = 36)	48.9 (29.1–67.4)	0.567	Yes (*n* = 27)	21.4 (13.4–37.4)	0.089
	No (*n* = 9)	51.6 (40.1–72.6)		No (*n* = 6)	46.0 (23.5–50.7)	

sCD30: soluble CD30; GN: glomerulonephritis; HD: hemodialysis; PD: peritoneal dialysis; m: months; *n*: number of patients investigated. ^∗^Median (interquartile range); *P* values: significance of differences between subgroups of patients.

**Table 3:** Outcomes of early and late posttransplant period.

Outcomes	Recipients, *n* = 45
Delayed graft function, *n* (%)	9 (20.0)^∗^
Slow graft function, *n* (%)	7 (15.6)
Acute rejection episodes during follow-up, *n* (%)	12 (26.7)
Very early (0–7th day), *n* (%)	5 (11.1)
Early (the 8th day–6 months), *n* (%)	4 (8.9)
Late (>6 months), *n* (%)	3 (6.7)
Pyelonephritis episodes during follow-up, *n* (%)	7 (15.6)
Early (the 8th day–2 months), *n* (%)	5 (11.1)
Late (>6 months), *n* (%)	2 (4.4)
Death with functioning graft during follow-up, *n* (%)	4 (8.9)
Graft failure during follow-up, *n* (%)	6 (13.3)

*n*: number of patients investigated. ^∗^Numbers (percentages).

**Table 4:** Median sCD30 levels according to initial graft function and presence of acute rejection and early pyelonephritis.

Groups of patients	*n*	Pretransplant sCD30	Posttransplant sCD30	*P* value
Initial graft function				
IGF	20	51.3 (34.5–70.6)^∗^	21.3 (14.6–41.3)	*P* < 0.001
SGF	5	27.3 (21.7–36.9)	19.9 (11.7–23.1)	*P* > 0.05
DGF	8	53.5 (39.7–72.9)	32.8 (25.7–39.9)	*P* = 0.012
Acute rejection				
Without AR	23	46.6 (30.8–70.6)	26.6 (12.4–40.1)	*P* < 0.001
AR at any time	11	47.1 (36.9–60.2)	21.3 (15.7–34.0)	*P* = 0.005
Very early AR (0–7th day)	5	56.1 (51.0–60.2)	21.2 (15.7–55.2)	*P* = 0.043
Early AR (the 8th day–6 months)	3	60.2 (43.3–70.7)	23.5 (21.4–56.7)	*P* = 0.108
Late AR (>6 months)	3	36.9 (17.1–40.9)	16.1 (9.2–34.0)	*P* = 0.109
Early pyelonephritis (the 8th day–2 months)				
No	29	49.5 (36.9–64.2)	26.6 (15.8–40.1)	*P* < 0.001
Yes	4	24.5 (16.7–49.0)	16.5 (11.2–22.2)	*P* = 0.068

sCD30: soluble CD30; IGF: immediate graft function; SGF: slow graft function; DGF: delayed graft function; AR: acute rejection; *n*: number of patients investigated. ^∗^Median (interquartile range).

**Table 5:** Significant predictors of eGFR at different time points of posttransplant period.

Predictive variables	Univariate linear regression			Multivariate linear regression		
	Beta	SE	*Р* value	Beta	SE	*Р* value
eGFR at 1 month (mL/min/1.73 m^2^)						
Deceased-donor graft	−0.333	0.144	0.025			
Impaired initial function	−0.660	0.115	<0.001	−0.616	0.115	<0.001
eGFR at 3 months (mL/min/1.73 m^2^)						
Recipient age	−0.370	0.143	0.013	−0.190	0.144	0.192
Pretransplant sCD30	0.313	0.147	0.038	0.145	0.141	0.309
Impaired initial function	−0.509	0.133	<0.001	−0.417	0.137	0.004
eGFR at 6 months (mL/min/1.73 m^2^)						
Recipient age	−0.341	0.145	0.024			
Pretransplant sCD30	0.316	0.146	0.037	0.291	0.131	0.032
Impaired initial function	−0.448	0.138	0.002	−0.378	0.129	0.005
Previous AR	−0.317	0.146	0.036	−0.366	0.127	0.006
eGFR at 1 year (mL/min/1.73 m^2^)						
Early AR	−0.321	0.150	0.038	−0.232	0.147	0.123
Serum creatinine at discharge	−0.355	0.148	0.021	−0.237	0.153	0.129
Deceased-donor graft	−0.325	0.150	0.036	−0.206	0.152	0.182
eGFR at 2 years (mL/min/1.73 m^2^)						
Posttransplant sCD30	0.417	0.178	0.027	0.382	0.161	0.026
Serum creatinine at discharge	−0.413	0.179	0.029	−0.342	0.167	0.052
Deceased-donor graft	−0.392	0.180	0.039	−0.232	0.169	0.184
eGFR at 3 years (mL/min/1.73 m^2^)						
AR at any time	−0.353	0.156	0.030	−0.197	0.141	0.172
Previous transplants	−0.489	0.145	0.002	−0.415	0.137	0.005
Serum creatinine at discharge	−0.396	0.153	0.014	−0.294	0.139	0.043
eGFR at 4 years (mL/min/1.73 m^2^)						
Serum creatinine at discharge	−0.432	0.155	0.008	−0.413	0.147	0.008
Pyelonephritis (8th day–2 months)	−0.349	0.167	0.037	−0.324	0.147	0.034
eGFR at 5 years (mL/min/1.73 m^2^)						
Serum creatinine at discharge	−0.389	0.156	0.018	−0.287	0.161	0.079
Second warm ischemia	−0.391	0.156	0.017	−0.292	0.161	0.083

eGFR: estimated glomerular filtration rate; beta: standardized regression coefficient; SE: standard error of beta; sCD30: soluble CD30; AR: acute rejection. ^∗^Only variables that significantly influenced the eGFR in univariate analysis are included.

## References

[1] Saran R., Li Y., Robinson B. (2014). US renal data system 2014 annual data report: epidemiology of kidney disease in the United States. *American Journal of Kidney Diseases*.

[2] Aguiar B., Santos Amorim T., Romãozinho C. (2015). Malignancy in kidney transplantation: a 25-year single-center experience in Portugal. *Transplantation Proceedings*.

[3] Israni A. K., Snyder J. J., Skeans M. A. (2010). PORT investigators. Predicting coronary heart disease after kidney transplantation: Patient Outcomes in Renal Transplantation (PORT) study. *American Journal of Transplantation*.

[4] Wang D., Wu W. Z., Chen J. H. (2010). Pre-transplant soluble CD30 level as a predictor of not only acute rejection and graft loss but pneumonia in renal transplant recipients. *Transplant Immunology*.

[5] Kim M. S., Kim H. J., Kim S. I. (2006). Pretransplant soluble CD30 level has limited effect on acute rejection, but affects graft function in living donor kidney transplantation. *Transplantation*.

[6] Shin J. H., Koo E. H., Ha S. H. (2016). The impact of slow graft function on graft outcome is comparable to delayed graft function in deceased donor kidney transplantation. *International Urology and Nephrology*.

[7] Halloran P. F., de Freitas D. G., Einecke G. (2010). An integrated view of molecular changes, histopathology and outcomes in kidney transplants. *American Journal of Transplantation*.

[8] Del Prete G. M., De Carli F., Almerigogna C. K. (1995). Preferential expression of CD30 by human CD4+ T cells producing Th2-type cytokines. *Federation of American Societies for Experimental Biology Journal*.

[9] Süsal C., Pelzl S., Döhler B., Opelz G. (2002). Identification of highly responsive kidney transplant recipients using pretransplant soluble CD30. *Journal of the American Society of Nephrology*.

[10] Pelzl S., Opelz G., Wiesel M. (2002). Soluble CD30 as a predictor of kidney graft outcome. *Transplantation*.

[11] Vondran F. W., Timrott K., Kollrich S. (2014). Pre-transplant immune state defined by serum markers and alloreactivity predicts acute rejection after living donor kidney transplantation. *Clinical Transplantation*.

[12] Halim M. A., Al-Otaibi T., Al-Muzairai I. (2010). Serial soluble CD30 measurements as a predictor of kidney graft outcome. *Transplantation Proceedings*.

[13] Slavcev A., Lácha J., Honsová E. (2005). Soluble CD30 and HLA antibodies as potential risk factors for kidney transplant rejection. *Transplant Immunology*.

[14] Matinlauri I. H., Kyllönen L. E., Salmela K. T., Helin H., Pelzl S., Süsal C. (2005). Serum sCD30 in monitoring of alloresponse in well HLA-matched cadaveric kidney transplantations. *Transplantation*.

[15] Weimer R., Süsal C., Yildiz S. (2005). sCD30 and neopterin as risk factors of chronic renal transplant rejection: impact of cyclosporine , tacrolimus, and mycophenolate mofetil. *Transplantation Proceedings*.

[16] Heinemann F. M., Rebmann V., Witzke O., Philipp T., Broelsch C. E., Grosse-Wilde H. (2007). Association of elevated pretransplant sCD30 levels with graft loss in 206 patients treated with modern immunosuppressive therapies after renal transplantation. *Transplantation*.

[17] Dong W., Shunliang Y., Weizhen W. (2006). Prediction of acute renal allograft rejection in early post-transplantation period by soluble CD30. *Transplant Immunology*.

[18] Valke L. L., van Cranenbroek B., Hilbrands L. B., Joosten I. (2015). Soluble CD30 does not predict late acute rejection or safe tapering of immunosuppression in renal transplantation. *Transplant Immunology*.

[19] Azarpira N., Aghdaie M. H., Malekpour Z. (2010). Soluble CD30 in renal transplant recipients: is it a good biomarker to predict rejection?. *Saudi Journal of Kidney Diseases and Transplantation*.

[20] Kamali K., Abbasi M. A., Farokhi B. (2009). Posttransplant soluble CD30 as a predictor of acute renal allograft rejection. *Experimental and Clinical Transplantation*.

[21] Chen Y., Tai Q., Hong S. (2012). Pretransplantation soluble CD30 level as a predictor of acute rejection in kidney transplantation: a meta-analysis. *Transplantation*.

[22] Abbas K., Muzaffar R., Zafar M. N., Mubarak M., Naqvi S. A., Rizvi S. A. (2009). Evaluation of pretransplant T-cell activation status by soluble CD 30 determination. *Journal of the Pakistan Medical Association*.

[23] Pelzl S., Opelz G., Daniel V., Wiesel M., Süsal C. (2003). Evaluation of posttransplantation soluble CD30 for diagnosis of acute renal allograft rejection. *Transplantation*.

[24] Spiridon C., Nikaein A., Lerman M., Hunt J., Dickerman R., Mack M. (2008). CD30, a marker to detect the high-risk kidney transplant recipients. *Clinical Transplantation*.

[25] Chen J. H., Lü R., Chen Y. (2005). Influence of pre-transplant serum level of soluble CD30 on the long-term survival rates of kidney transplant recipients and grafts. *Zhonghua Yi Xue Za Zhi*.

[26] Fernández-Ruiz M., Parra P., López-Medrano F. (2017). Serum sCD30: a promising biomarker for predicting the risk of bacterial infection after kidney transplantation. *Transplant Infectious Disease*.

[27] Wang D., Wu W., Yang S., Wang Q., Tan J. (2012). Post-transplant monitoring of soluble CD30 level as predictor of graft outcome: a single center experience from China. *Transplant Immunology*.

[28] Amirzargar M. A., Amirzargar A., Basiri A. (2014). Early post-transplant immune monitoring can predict long-term kidney graft survival: soluble CD30 levels, anti-HLA antibodies and IgA-anti-Fab autoantibodies. *Human Immunology*.

[29] Süsal C., Döhler B., Ruhenstroth A. (2016). Donor-specific antibodies require preactivated immune system to harm renal transplant. *eBioMedicine*.

[30] Melendreras S. G., Martínez-Camblor P., Menéndez A. (2014). Soluble co-signaling molecules predict long-term graft outcome in kidney-transplanted patients. *PloS One*.

[31] Delgado J. C., Pavlov I. Y., Shihab F. S. (2009). Post-transplant increased levels of serum sCD30 is a marker for prediction of kidney allograft loss in a 5-year prospective study. *Transplant Immunology*.

[32] Süsal C., Döhler B., Sadeghi M. (2011). Posttransplant sCD30 as a predictor of kidney graft outcome. *Transplantation*.

[33] Kovac J., Arnol M., Vidan-Jeras B., Bren A. F., Kandus A. (2008). Does pretransplant soluble CD30 serum concentration affect deceased-donor kidney graft function 3 years after transplantation?. *Transplantation Proceedings*.

[34] Iv R., He Q., Wang H. P., Jin J., Chen Y., Chen J. H. (2008). High serum level of the soluble CD30 identifies Chinese kidney transplant recipients at high risk of unfavorable outcome. *Transplantation Proceedings*.

[35] Weimer R., Süsal C., Yildiz S. (2006). Post-transplant sCD30 and neopterin as predictors of chronic allograft nephropathy: impact of different immunosuppressive regimens. *American Journal of Transplantation*.

[36] KDIGO (2013). KDIGO 2012 clinical practice guideline for the evaluation and management of chronic kidney disease. *Kidney International Supplements*.

[37] Sánchez-Fructuoso A., Naranjo Garcia P., Calvo Romero N. (2007). Effect of the brain-death process on acute rejection in renal transplantation. *Transplantation Proceedings*.

[38] Sun Q., Liu Z. H., Ji S. (2006). Late and early C4d-positive acute rejection: different clinico-histopathological subentities in renal transplantation. *Kidney International*.

[39] Lopes D., Barra T., Malheiro J. (2015). Effect of different sensitization events on HLA alloimmunization in kidney transplantation candidates. *Transplantation Proceedings*.

[40] Vaidya S., Partlow D., Barnes T., Gugliuzza K. (2006). Pretransplant soluble CD30 is a better predictor of posttransplant development of donor-specific antibodies and acute vascular rejection than panel reactive antibodies. *Transplantation*.

[41] Rajakariar R., Jivanji N., Varagunam M. (2005). High pre-transplant soluble CD30 levels are predictive of the grade of rejection. *American Journal of Transplantation*.

[42] Schaefer S. M., Süsal C., Opelz G. (2016). Pre-transplant soluble CD30 in combination with total DSA but not pre-transplant C1q-DSA predicts antibody-mediated graft loss in presensitized high-risk kidney transplant recipients. *HLA*.

[43] Rodríguez L. M., París S. C., Arbeláez M. (2007). Kidney graft recipients with pretransplantation HLA class I antibodies and high soluble CD30 are at high risk for graft loss. *Human Immunology*.

[44] Altermann W., Schlaf G., Rothhoff A., Seliger B. (2007). High variation of individual soluble serum CD30 levels of pre-transplantation patients: sCD30 a feasible marker for prediction of kidney allograft rejection?. *Nephrology, Dialysis, Transplantation*.

[45] Kanter Berga J., Pallardo Mateu L. M., Beltran Catalan S. (2011). Donor-specific HLA antibodies: risk factors and outcomes after kidney transplantation. *Transplantation Proceedings*.

[46] Lebranchu Y., Baan C., Biancone L. (2014). Pretransplant identification of acute rejection risk following kidney transplantation. *Transplant International*.

[47] Campbell S. B., Hothersall E., Preston J. (2003). Frequency and severity of acute rejection in live- versus cadaveric-donor renal transplants. *Transplantation*.

[48] Loverre A., Divella C., Castellano G. (2011). T helper 1, 2 and 17 cell subsets in renal transplant patients with delayed graft function. *Transplant International*.

[49] Requião-Moura L. R., de Durão Junior M. S., Matos A. C., Pacheco-Silva A. (2015). Ischemia and reperfusion injury in renal transplantation: hemodynamic and immunological paradigms. *Einstein (Sao Paulo)*.

[50] Espinar M. J., Miranda I. M., Costa-de-Oliveira S., Rocha R., Rodrigues A. G., Pina-Vaz C. (2015). Urinary tract infections in kidney transplant patients due to Escherichia coli and Klebsiella pneumoniae-producing extended-spectrum β-lactamases: risk factors and molecular epidemiology. *PloS One*.

[51] Donders G. G. (2002). Lower genital tract infections in diabetic women. *Current Infectious Disease Reports*.

[52] Kusanovic J. P., Romero R., Esoinoza J. (2007). Maternal serum soluble CD30 is increased in pregnancies complicated with acute pyelonephritis. *The Journal of Maternal-Fetal & Neonatal Medicine*.

[53] Kim H. M., Shin H. Y., Jeong H. J. (2000). Reduced IL-2 but elevated IL-4, IL-6, and IgE serum levels in patients with cerebral infarction during the acute stage. *Journal of Molecular Neuroscience*.

[54] Guo S., Zhao J. (2012). Immunotherapy for tuberculosis: what’s the better choice?. *Frontiers in Bioscience (Landmark Edition)*.

[55] Tan T. T., Coussens L. M. (2007). Humoral immunity, inflammation and cancer. *Current Opinion in Immunology*.

[56] Feng Y., Yin H., Mai G. (2011). Elevated serum levels of CCL17 correlate with increased peripheral blood platelet count in patients with active tuberculosis in China. *Clinical and Vaccine Immunology*.

